# Improving transitions and outcomes of sepsis survivors (I-TRANSFER): a type 1 hybrid protocol

**DOI:** 10.1186/s12904-022-00973-w

**Published:** 2022-06-02

**Authors:** Melissa O’Connor, Erin E. Kennedy, Karen B. Hirschman, Mark E. Mikkelsen, Partha Deb, Miriam Ryvicker, Nancy A. Hodgson, Yolanda Barrón, Michael A. Stawnychy, Patrik A. Garren, Kathryn H. Bowles

**Affiliations:** 1 M. Louise College of Nursing, Villanova University, 800 Lancaster Avenue, Villanova, PA 19085 USA; 2 Fellow, Betty Irene Moore Fellowship for Nurse Leaders and Innovators, Sacramento, CA USA; 3 School of Nursing, NewCourtland Center for Transitions in Health, University of Pennsylvania, 418 Curie Boulevard, Philadelphia, PA 19104 USA; 4 Pulmonary, Allergy, and Critical Care Division, Department of Medicine, Perelman School of Medicine, University of Pennsylvania, 3400 Civic Center Boulevard, Philadelphia, PA 19104 USA; 5 Department of Economics, Hunter College, 695 Park Avenue, New York, 10065 USA; 6 Center for Home Care Policy & Research, VNS Health, 220 East 42nd Street, New York, NY 10017 USA

**Keywords:** Sepsis, Home health care, Follow up physician visits, Skilled nursing visits, Palliative care, Implementation science, Type 1 hybrid, Transitions in care

## Abstract

**Background:**

This protocol is based on home health care (HHC) best practice evidence showing the value of coupling timely post-acute care visits by registered nurses and early outpatient provider follow-up for sepsis survivors. We found that 30-day rehospitalization rates were 7 percentage points lower (a 41% relative reduction) when sepsis survivors received a HHC nursing visit within 2 days of hospital discharge, at least 1 more nursing visit the first week, and an outpatient provider follow-up visit within 7 days compared to those without timely follow-up. However, nationwide, only 28% of sepsis survivors who transitioned to HHC received this timely visit protocol. The opportunity exists for many more sepsis survivors to benefit from timely home care and outpatient services. This protocol aims to achieve this goal.

**Methods:**

Guided by the Consolidated Framework for Implementation Research, this Type 1 hybrid pragmatic study will test the effectiveness of the Improving Transitions and Outcomes of Sepsis Survivors (I-TRANSFER) intervention compared to usual care on 30-day rehospitalization and emergency department use among sepsis survivors receiving HHC. The study design includes a baseline period with no intervention, a six-month start-up period followed by a one-year intervention period in partnership with five dyads of acute and HHC sites. In addition to the usual care/control periods from the dyad sites, additional survivors from national data will serve as control observations for comparison, weighted to produce covariate balance. The hypotheses will be tested using generalized mixed models with covariates guided by the Andersen Behavioral Model of Health Services. We will produce insights and generalizable knowledge regarding the context, processes, strategies, and determinants of I-TRANSFER implementation.

**Discussion:**

As the largest HHC study of its kind and the first to transform this novel evidence through implementation science, this study has the potential to produce new knowledge about the impact of timely attention in HHC to alleviate symptoms and support sepsis survivor’s recovery at home. If effective, the impact of this intervention could be widespread, improving the quality of life and health outcomes for a growing, vulnerable population of sepsis survivors. A national advisory group will assist with widespread results dissemination.

## Background

Sepsis is a life-threatening acute organ dysfunction secondary to infection [1]. Acute care hospitals in the United States (US) discharge over 1.5 million sepsis survivors annually [1–4]. Across diverse healthcare systems, 30-day hospital readmission rates are consistently high for survivors [5–9]. Sepsis is a serious illness and survivors are vulnerable to substantial post-discharge morbidity and mortality [10], with readmission rates rivaling those for heart failure, pneumonia, and myocardial infarction [5, 11, 12]. Annual health care costs for sepsis survivors are nearly double the costs of these other conditions [12]. Sepsis survivors are twice as likely as non-sepsis patients to be readmitted by 30 days [12], often with a new or recurrent infection [13] with 32% of these 30-day readmissions occurring within 7 days [6, 14], and in-patient costs alone ranging $23.7 [15] to $27 billion per year [16] making sepsis the costliest inpatient diagnosis in the US [17]. When hospitalized, these vulnerable, mainly older adults, are exposed to increased risk for hospital-acquired infections and medical errors [18]. Sepsis survivors often experience impaired functional status [19], reduced quality of life [20], and accelerated cognitive decline [20], all of which are associated with a shortened life span [21]. A subset of sepsis survivors, especially those who experience these complex sequelae, may greatly benefit from goals of care discussions and palliative care [22, 23].

Annually, up to one-third of sepsis survivors transition from acute care to skilled home health care (HHC) where nurses monitor for reinfection, support uninterrupted medication management, manage symptoms, and work with patients, caregivers, and other providers to support continued recovery [6, 24]. Vigilant, timely community-based monitoring to address subtle changes in symptoms, conduct medication reconciliation, and reinforce the plan of care is necessary to prevent avoidable hospital admissions and keep patients at home. While as many as one-third of sepsis survivors are prescribed HHC after hospital discharge [24], there is wide variability in the intensity and timing of HHC visits for this vulnerable population [25]. Medicare Conditions of Participation require that HHC agencies conduct the first home visit within two days of referral or the patient’s return home, or on the physician-ordered start of care date. Therefore, agencies typically have considerable latitude in determining the timing and volume of services provided [25–28]. Agencies may measure timeliness of the first visit by when they “accept” the referral, versus when received or by facility discharge date. In doing so, patients may not be seen for more than two days after discharge, as our prior work indicates.

It is during the first few weeks of HHC that sepsis survivors are most at risk for rehospitalization [14], indicating the need for timely attention to symptom management afforded by HHC services following hospital discharge. Frontloading, defined as early and intensive nursing visits and endorsed by The National Association for Homecare & Hospice [29, 30], Visiting Nurse Associations of America [31], The Home Health Quality and Improvement National Campaign [32], and The Institute for Healthcare Improvement [33], has been recommended as an industry standard for decades. While 64% of HHC agencies claim to be utilizing frontloading [30], our national Medicare claims studies found that only 23% of heart failure [34], 44.7% of sepsis [24] and 39% of patients overall [34] received frontloaded nursing visits when the timing was measured from the hospital discharge date. Our results indicate that while frontloading has been promoted as a best practice for high-risk populations, it is not widely or consistently implemented across HHC agencies nationally.

The first homecare visit, usually conducted by a registered nurse, is one of the most critical steps of the home care episode [35, 36], and is important for continuity of care [18, 37, 38]. Too often the HHC nurse conducts the admission visit with fragmented, incomplete, or inaccurate knowledge of the patient’s clinical condition [39, 40]. In our prior work, among sepsis survivors readmitted by 30-days, 33% of hospital readmissions occurred within 7 days. Moreover, we found a mismatch between the hospital diagnoses and the HHC diagnoses, indicating HHC clinicians may not know the patient is a sepsis survivor [14]. For hundreds of thousands of sepsis survivors, the most frequent diagnoses in HHC were pneumonia and urinary tract infection, with sepsis ICD codes appearing only 4% of the time [14]. Further, most of the 7-day readmission risk factors identified by our team were noted after the HHC admission [14]. It is too late to wait for the HHC assessment to occur on the first home visit (which could occur several days after hospital discharge and be available to others in the record up to 5 days later). Providing high quality information from the acute care referral source to increase awareness of the sepsis survivors facilitates accurate care planning and supports provision of earlier visits, higher quality care, and prevention of avoidable, early rehospitalization for a sizable and increasing population of sepsis survivors [41–44]. The National Consensus Project Clinical Practice Guidelines for Quality Palliative Care support access to palliative care throughout the trajectory of severe illnesses like sepsis, increasingly in community or home settings. Improving the transfer of information can increase access to these resources, which can enable patients and families to receive support in their homes [45]. For these reasons, our intervention starts in acute care to improve identification of the sepsis survivor and to highlight the diagnosis for HHC clinicians.

Physicians must certify the eligibility of patients for HHC, and review and sign the orders for care [46]. Currently, there is no requirement that the physician ordering HHC must see the patient other than the relatively new requirement for a single face-to-face encounter that can be up to 90 days before or 30 days after initiation of HHC services. In general, early outpatient provider visits for patients discharged from acute care vary in timing from 2 days [47] to 30 days [48] and are most effective for those at highest risk [49]. Patients are more likely to attend an outpatient provider visit if scheduled before leaving the hospital [47]. Despite this evidence, our experience is that patients are often expected to schedule outpatient visits themselves. In our studies, for home health patients, outpatient provider follow-up alone, within 7-days, without nursing visits occurred only 24.2% [50] of the time for heart failure patients and 11% of the time for sepsis survivors [24].

Our prior evidence indicates that neither early HHC nursing nor outpatient provider visits alone significantly reduced hospital readmissions in two high-risk populations (heart failure and sepsis). In contrast, when the two practices were combined, early hospital readmissions were significantly reduced. In our prior comparative effectiveness study using national Medicare data and an instrumental variable analysis approach, we found 30-day rehospitalization rates were 7 percentage points lower (41% relative reduction) for sepsis patients when a HHC nursing visit was received within 2 days of hospital discharge, at least 1 more nursing visit the first week, and an outpatient provider follow-up visit by 7 days occurred compared to those without timely follow-up [14, 24].

The literature suggests a critical need to implement best practices to increase awareness of sepsis survivors and to improve the delivery of timely care during transitions [14, 39–43]. However, no previous studies have targeted the sepsis survivor in HHC. Based on the high impact of our prior work, we are investigating the effectiveness and implementation of the Improving Transitions and Outcomes of Sepsis Survivors (I-TRANSFER) intervention to increase the proportion of sepsis survivors receiving this powerful combination of two best practices. To advance the science of transitional care for sepsis survivors, this protocol will test the effectiveness of these practices in the real world and study its implementation with a pragmatic, multi-center, Type 1 hybrid design in partnership with five dyads of acute care and HHC providers. As the largest study of its kind in HHC, it will produce new knowledge about the real-world effectiveness and implementation determinants; and will focus on an understudied process (transition to and care in HHC) and an understudied population (sepsis survivors) to tackle a large, costly, and common rehospitalization challenge where little evidence exists. Many HHC agencies do not have the resources to study how to optimize transition of care processes. Therefore, our study findings will bring process and structure to a very common transition procedure and inform the HHC industry with knowledge that could apply to other disease states. If shown to be effective, information on context, barriers likely to be encountered, other determinants, and the most feasible strategies for implementation will inform the hospital and HHC industries for widespread implementation of an effective practice.

Our I-TRANSFER intervention is designed to address several barriers and gaps that may jeopardize the implementation of early post-acute surveillance. Sepsis survivors are not consistently identified with a medical diagnosis of sepsis until after discharge from acute care [51–55]. This results in the diagnosis and other patient characteristics not being adequately communicated to the next level of care [1, 39, 40, 56]. Home health clinicians likely do not know they are receiving a sepsis survivor. Second, there is little evidence to guide the transition of sepsis survivors to HHC. Our protocol is designed to provide a critically needed, especially post-pandemic, best practice intervention model to clinicians and the HHC industry nationwide. This protocol is even more timely given the pandemic, as research suggests that COVID-19 can lead to sepsis [57] and many hospitalized patients prefer to recover at home due to skilled nursing facility capacity and safety concerns [58]. Third, among sepsis survivors transitioned to HHC, and not readmitted within the first week, only 28% received the early visit protocol, shown by our team to be associated with significantly reduced readmissions [24]. This presents an important opportunity to generate further evidence of effectiveness and inform widespread implementation to help many more sepsis survivors. Our advisory group, comprised of national leaders in sepsis care and HHC professional organizations will facilitate widespread dissemination of our findings, from academic nursing and medicine to industry practitioners.

## Methods

### Study aims and hypothesis

#### Aim 1, effectiveness of the I-TRANSFER intervention

Aim 1 of our study is to test the effectiveness of the I-TRANSFER intervention compared to usual care on 30-day rehospitalization and emergency department (ED) use among sepsis survivors receiving HHC. We hypothesize that compared to usual care, sepsis survivors who receive the I-TRANSFER intervention will have significantly fewer all-cause 30-day rehospitalizations, inpatient days if rehospitalized, and ED visits within 30 days of hospital discharge.

#### Aim 2, I-TRANSFER implementation

Aim 2 of our study is to produce insights and generalizable knowledge regarding the context, processes, strategies, and determinants of I-TRANSFER implementation. We will assess readiness of the sites using the Organizational Readiness for Implementing Change (ORIC) survey. The ORIC survey includes 12 questions related to the participant’s perception of their organization’s readiness to implement the intervention including characteristics like confidence, motivation, support, flexibility, addressing challenges, and coordination [59]. We will study the implementation using qualitative methods guided by the Consolidated Framework for Implementation Research (CFIR) [60]. We will also test two process hypotheses. First, compared to usual care, sepsis survivors receiving I-TRANSFER will have sepsis identified as a HHC diagnosis on the Outcomes Assessment Information Set (OASIS) significantly more often. Second, sepsis survivors receiving I-TRANSFER will receive early and intense HHC nursing visits (within 2 days of hospital discharge + 1 more that week) and community provider visits by 7-days more often. The OASIS is a comprehensive assessment tool, mandated by the Centers for Medicare and Medicaid (CMS), designed to collect nearly 100 items related to a home health recipient’s functional status, clinical status, and service needs at several time points during a HHC episode [34].

### Conceptual framework

#### Aim 1, effectiveness of the I-TRANSFER intervention

**Fig. 1:**
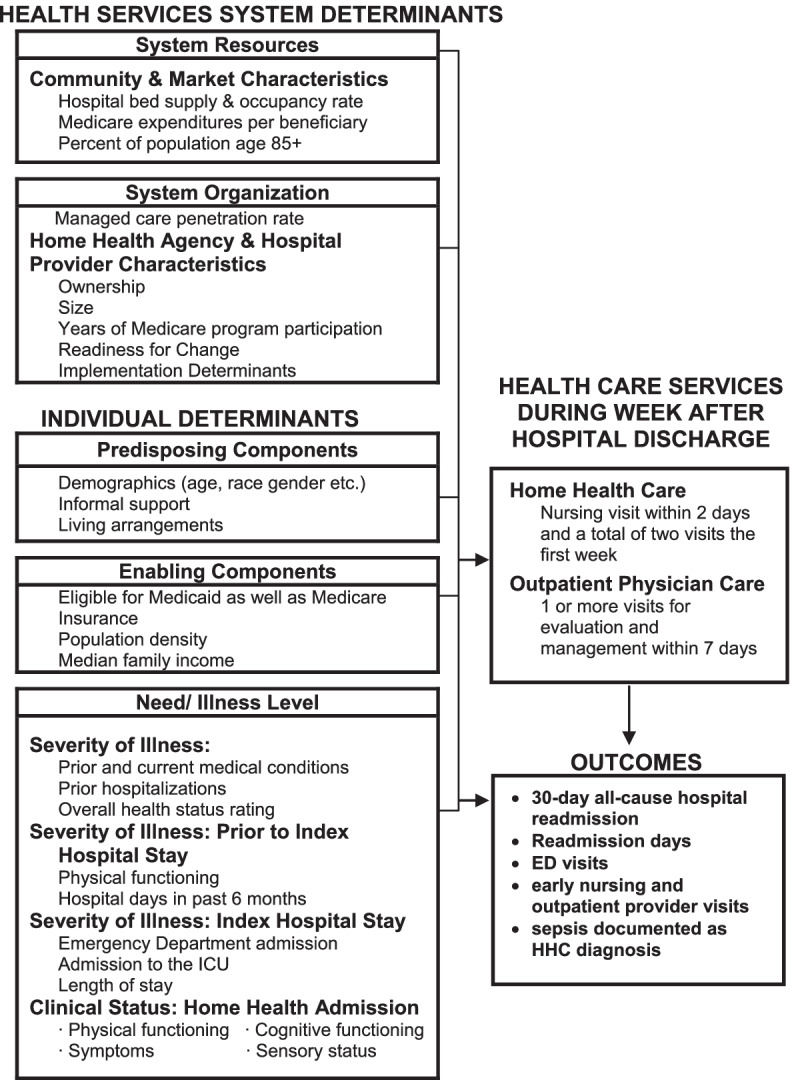
Andersen Behavioral Model of Health Services. Health services system determinants

As illustrated in Fig. 1, the Andersen Behavioral Model of Health Services [61] will guide Aim 1. Service use is modeled as a function of health care system factors, and individual determinants. Health services system determinants include resource and organizational factors. Community and market characteristics (e.g., the supply of HHC agencies, physicians, and other health services) are some examples of system resources. Organizational factors include HHC agency and hospital characteristics such as whether the agency is owned by a hospital, part of a chain, or it operates as a for-profit, nonprofit or government entity. The Organizational Readiness for Implementing Change (ORIC) survey results will be a factor in this domain. Implementation determinants (barriers, enablers, strategies) from the CFIR domains discovered in Aim 2 may also be added to the model as variables to understand their impact on the outcomes.

There are three types of Individual Determinants in the Andersen framework. Predisposing factors include age, sex as a biological variable, race, and informal support, for example. Enabling factors include facilitators of the use of health care services (e.g., health insurance, income). Need/illness level includes health status such as acute and chronic disease, cognitive and physical functioning, symptoms such as pain and anxiety, and recent prior hospitalizations. Determinants in the Andersen framework will also be considered for risk adjustment and when forming our national, covariate-balanced control sample (Aim 2). Conceptually, the outcomes are jointly determined by the determinants in the Andersen framework as well as the home health and physician services provided during the HHC episode. Because sicker patients are more likely to use more HHC and physician services and are likely to have worse outcomes – that is, the receipt of increasing levels of HHC and physician services is endogenous – a simple comparison of outcomes will yield misleading biased results and are taken into consideration in the analytic plan.

#### Aim 2, I-TRANSFER implementation

The Consolidated Framework for Implementation Research (CFIR) guides Aim 2 and contains 5 constructs to assess context and implementation determinants at each site. Context has been defined as the set of circumstances or unique factors that surround a particular implementation effort [60] and anything external to the intervention which impedes or strengthens its effects [5, 62]. Determinants are factors that might prevent or enable practice change including barriers, enablers, incentives or disincentives [60, 63]. Processes and strategies are the workflows and interventions applied to address determinants and achieve the implementation [63].

### Study design

The study was designed based on evidence from our previous study; extensive preliminary work with national data, hospital, and HHC stakeholders; & implementation science literature [24, 25, 34, 50, 60, 64–72]. The study is a pragmatic, Type 1 hybrid design, that primarily evaluates effectiveness via Aim 1, with an observational, implementation component integrated into its design via Aim 2. This novel research design is being increasingly used in the evaluation of service delivery type interventions, especially when conditions are heterogeneous and traditional randomization of individuals is not possible [73]. This pragmatic design involves an initial period in which no sites are exposed to the intervention followed by a six-month onboarding period, a 12-month intervention period, and a 6-month maintenance period. In the initial proposal we planned a stepped wedge cluster randomized trial but due to the COVID-19 pandemic the 2020 cohort are unsuitable as a comparison cohort requiring extension of the inclusion dates for the comparison dataset to 2021–2024, and staggered rollout required by stepped wedge design is no longer feasible to finish the study on time.

Sepsis survivors transitioned to the participating HHC agencies during the period before implementation serve as the usual care control group compared to those exposed to I-TRANSFER, the intervention group. To address historical threats and to increase generalizability, we form control comparison groups from national Medicare clinical and administrative claims data within and outside our partner sites to compare to our intervention group: 1) one year prior to any contact with our team; 2) during the onboarding period, and 3) across the entire study period. Observations on survivors from outside our partners’ sites will be weighted using entropy balancing [74–76] to produce a covariate-balanced comparison group.

Each hospital and HHC agency dyad will assemble a site implementation team comprised of key stakeholders and project champions involved in the planning and implementation. The principal investigator and at least two other research team members will visit each site in person and stay in close phone or email contact with the site coordinator and team for onboarding and Aim 2 data collection. Additional contacts may occur if needed. We have planned 12 in-person visits and frequent, standardized phone, virtual and/or email contacts. Expected site stakeholders may include, for example, a programmer to write a query to identify the inpatient sepsis survivors; hospitalists, emergency room physicians, sepsis experts, and ambulatory providers to determine follow-up appointment workflow, and hospital case managers are expected to be important informants overall. On the HHC side, the teams will include a hospital based HHC liaison who facilitates the referrals and transfer of patient information to the HHC agency; an HHC intake nurse who schedules the first visit and assigns the visiting nurses; a HHC nurse who knows the workflow for planning visits and working with patients; an intake director who understands the entire workflow; the Directors of Nursing or Chief Operating Officers who oversee operations. These stakeholders will participate in individual, or group interviews and survey completion as described below. An estimated 60 stakeholders will provide informed consent prior to data collection. A study site coordinator identified by each dyad will assist with communication, scheduling, oversight, and data collection at each site. A project manager will track progress and support the work across all sites. Stakeholder consent to participate will be obtained prior to each interview by the study team member conducting the interview.

Implementation of the I-TRANSFER components will be guided by the stakeholders and research team using intervention mapping, as described below. The context and determinants (barriers, enablers), processes, and strategies encountered during Aim 1 will be collected for Aim 2 analysis. Hospital partners will work with the study and HHC team to achieve the following components of I-TRANSFER: identify the sepsis survivors during the hospitalization, make the outpatient follow-up appointment before discharge, notify the HHC agency of a sepsis survivor, conduct timely home health visits and outpatient visits in week one. Based on our preliminary work, we have identified several effective strategies that we can suggest to better identify the sepsis survivor, including generalizable use of sepsis order sets within the electronic health record [14]. We will also recommend use of the Centers for Disease Control (CDC) Epicenters Sepsis Clinical Surveillance Definition, which has the advantage of identifying confirmed cases of sepsis given the criteria of sustained antibiotic delivery [35, 77, 78]. We will examine fidelity to these recommended components as potential implementation enablers.

Challenges we expect to face in making and achieving a timely outpatient visit are limited appointment availability, and patients who cancel or do not attend the visit. A potential strategy is to increase awareness about I-TRANSFER’s evidence and include a script for the clinician to educate the patient about the value and importance of the outpatient visit. Transportation may be an issue as well and will be brainstormed for solutions with the stakeholders and tracked as a barrier. Hospital based personnel will notify the HHC intake department when the patient is a sepsis survivor. The intervention mapping will determine the workflow for this component with each site. It may include phone calls, text messaging, faxed or electronic documents.

HHC intake personnel will likely schedule the nurse visits and notify the visiting nurse of the sepsis diagnosis and date of the outpatient appointment. To meet protocol, the first registered nurse home visit will occur within 2 days of hospital discharge, with a second visit that week. The patient will be encouraged and assisted if necessary (e.g. transportation) to attend their scheduled outpatient appointment.

A four-member interdisciplinary, national advisory board with expertise in sepsis and HHC will advise the team via teleconference once in year one and twice in years 2–5. They will review the CFIR interview guide, and findings from the onboarding and implementation phases, providing advice as the study progresses. The national advisory board brings expertise in sepsis and HHC and will have a major role in facilitating dissemination of the study findings through access to national stakeholder groups.

### Study period

Onboarding is a 6-month period when the implementation and research teams plan the implementation. Intervention is the 12-month period when I-TRANSFER is in operation. Maintenance is a 6-month period when the research team assesses whether the intervention continues without research team monitoring and feedback. The analysis will be an intent to treat. We will adjust for time, or any health system interventions, as well as patient covariates that may confound the findings using generalized mixed models.

### Study sample

#### Aim 1, effectiveness of the I-TRANSFER intervention

Sample sizes needed to provide sufficient power were calculated using Stata [79]. Preliminary unpublished data of a national sample show on average a 23% 30-day readmission rate for sepsis survivors in HHC. Our previous study showed a 7-percentage point or 41% relative reduction among those receiving early visits [24], and other studies report that 22–45% of readmissions are preventable [80]. Therefore, to detect a 7-point reduction (30.4% relative decrease) in the readmission rate under the intervention we would require an average of 400 patients per time interval. Current estimates from the sites give us 869 per period.

The I-TRANSFER intervention is targeted at the hospital and HHC agency and is considered part of clinical care implemented for all sepsis survivors. There is no individual patient recruitment, prospective assignment, or primary data collection from patients. The analysis will be completed on existing datasets. We will analyze a sample drawn from all Medicare fee-for-service sepsis survivors discharged to HHC during a 4-year period (01/01/2021 and 12/31/2024). Analyses from our 2013–14 dataset suggests that without exclusions there will be approximately 800,000 sepsis discharges per year who receive HHC [24].

Ten clinical sites located in the United States have committed to participate as dyads, 5 referring hospitals and their 5 partnered home health agencies. Sites were selected based on our relationships with staff, ownership status (hospital owned or free-standing), commitment to appoint an implementation team, size, geography, and diversity (academic and community). We sought regional diversity with sites in the East and West. To be eligible HHC agencies must be Medicare certified to generate claims and confirm a hospital referral source as a partner.

#### Aim 2, I-TRANSFER implementation

We expect approximately 60 stakeholders to comprise the Aim 2 sample. Acute care and HHC site champions including hospitalists, emergency room physicians, programmers, managers, sepsis experts, ambulatory providers, care coordinators, Directors of Nursing, home health liaisons, intake personnel and nurses, are the expected subjects. After informed consent, they will be administered the ORIC survey and interviewed by the study team using the CFIR interview guide.

### Measures and outcomes

#### Aim 1, effectiveness of the I-TRANSFER intervention

The primary outcome of Aim 1 is 30-day all-cause hospital readmissions. Secondary outcomes are number of inpatient days (if rehospitalized), and ED use within 30 days, all measured through the Medicare Administrative Data.

#### Aim 2, I-TRANSFER implementation

The outcomes of Aim 2 will be the alternative approaches in workflow, barriers, facilitators, and differences in acceptability observed between the study sites during implementation of the I-TRANSFER protocol. These alternative approaches will be noted, compared, contrasted, and reported as study results so agencies with similar barriers can see suggested solutions to increase generalizability of the findings.

### Data sources and collection

#### Aim 1, effectiveness of the I-TRANSFER intervention

A national sample of Medicare administrative, home health OASIS and claims data from CMS Chronic Condition Warehouse (CCW) will be employed (Table 1). This will yield detailed information about the index hospital stay, subsequent HHC and outpatient provider services and outcomes. It also will provide information about the characteristics of hospitals and admitting HHC agencies, and the health care market areas where patients live. The CCW maintains calendar year files so we will request files between 01/01/2021 and 12/31/2024. We will request CCW data on all Medicare beneficiaries with a claim for a HHC service provided one year prior to the start of the study to 6 months after the end. Within these data sets, we will identify the sepsis survivors transitioned from participating hospitals to their partner HHC agency by merging the Provider of Service File with the claim files. The additional sepsis survivors found in the CCW data, not associated with our participating institutions, will serve as additional controls.

**Table 1:** Variables Obtained from the Medicare Claims and OASIS Home Health Admission Files

Data	Source
*Physician, Home Health Agency and Hospital Characteristics*	
Physician specialty	Downloadable National Provider Identifier File (CMS)
Home Health Agency and Hospital Ownership	Provider of Services File
Home Health Agency and Hospital Control/auspices (for- profit/non-profit/public)	Provider of Services File
Size of home health agency, # of annual admissions	Provider of Services File
*Beneficiary Sociodemographic, Clinical and Cost Variables*	
Demographics	Medicare Beneficiary Summary File; OASIS
Informal support	OASIS
Living arrangements	OASIS
Eligible for Medicare and Medicaid	Medicare Beneficiary Summary File
Health care use and cost (ie. Medicare program payments)	MedPAR/Medicare Parts A and B SAF claims
Medical diagnoses ICD codes, acute and HHC	MedPAR/Medicare Parts A and B SAF claims; OASIS
Index hospital length of stay	MedPAR
Cognitive, physical, sensory function	OASIS
*Early Nursing and Outpatient Provider Visits Following Index Hospital Discharge*	
Timing and number of home health visits	Medicare Home Health SAF claims
Timing of first physician, physician assistant or nurse practitioner visit for outpatient evaluation & mgt	Mediare Part B SAF claims
*Beneficiary Readmission Outcomes, Mortality and Other Outcomes after Home Health Admission*	
Timing and number of Medicare inpatient hospitalizations and ED use	MedPAR
Inpatient admission reason	MedPAR
Cognitive, physical, sensory function	OASIS
*Early Nursing and Outpatient Provider Visits Following Index Hospital Discharge*	
Timing and number of home health visits	Medicare Home Health SAF claims
Timing of first physician, physician assistant or nurse practitioner visit for outpatient evaluation & mgt	Mediare Part B SAF claims
*Beneficiary Readmission Outcomes, Mortality and Other Outcomes after Home Health Admission*	
Timing and number of Medicare inpatient hospitalizations and ED use	MedPAR
Inpatient admission reason	MedPAR

Source files will also include: (1) the CMS Provider of Services file containing information on hospital, home health, and other provider characteristics such as ownership and length of participation in the Medicare and Medicaid programs; (2) the Area Health Resources file which is a national, county-level record of the supply of health care services maintained by the US Health Resources and Services Administration; and (3) Census data that include ZIP-code level socio-economic measures for the patients’ place of residence.

The OASIS is a comprehensive assessment tool designed to collect nearly 100 items related to a home health recipient’s functional status, clinical status, and service needs at several time points during a HHC episode. Mandated by CMS since 1999, the OASIS is the most comprehensive national data set on HHC patients. OASIS data are collected upon admission, every 60 days, if transferred to an inpatient facility, and at discharge. The OASIS will be the source of detailed patient clinical data including, importantly, functional status, health therapies, the result of a drug regimen review and whether patient-specific parameters have been established for notifying the physician of changes in vital signs or other clinical findings. The reliability of OASIS functional items varies. Mobility and transferring have Kappa values > 0.70, while lower body dressing and bathing have Kappa values of 0.61 and 0.53, respectively. All other items to be analyzed in the proposed study have at least moderate reliability (Kappa ≥ 0.40) with the exception of eating and meal preparation (Kappa = 0.38 for both) which primarily will be used to control for differences in functioning on HHC admission [80].

#### Aim 2, I-TRANSFER implementation

Data for Aim 2 will be generated through three sources: by administering the Organizational Readiness for Implementing Change (ORIC) survey, a needs assessment at each site, and the Consolidated Framework for Implementation Research (CFIR) interviews.

### Organizational Readiness for Implementing Change (ORIC) survey

At baseline, each acute care and home health stakeholder will complete the ORIC 12-item survey to assess readiness for change that refers to organizational members' shared beliefs in their collective capability to change (change efficacy, items 1,3,5,7,9,11) and their shared resolve to implement a change (change commitment, items 2,4,6,8,10,12). Based on a 5-point Likert scale of agree to disagree, the tool has high inter-item consistency, inter-rater reliability, inter-rater agreement, good model fit and item loadings. The ORIC score is calculated by summing across all items. Lower scores represent less organizational readiness for implementing change; higher scores represent a more favorable readiness for implementing change [59].

Implementation Mapping. The systematic process of implementation mapping has 5 steps for developing strategies to improve adoption, implementation, and maintenance of evidence-based interventions in the real world [64]. These steps will be informed by the needs assessments, the CFIR interviews with stakeholders and the ORIC survey. Step 1 calls for an implementation needs assessment of eight objectives to identify barriers and enablers, adopters, and implementers (Table 2). At the end of this step, we will know who the champions will be, who will make resources available, and who are the decision makers. In Step 2, together with the dyads, we generate adoption and implementation outcomes and performance objectives, identify determinants, and create matrices of change objectives. This step determines who must do what and how will success be measured. For example, the hospital case managers may be tasked with determining the workflow and strategies for making the follow-up outpatient appointment and educating sepsis survivors on its importance. Matrices of change objectives answer what must change to make something happen [64]. Each performance objective related to I-TRANSFER (e.g., identify the survivors, communicate with HHC, make the appointment, etc.) and its determinants, identified in earlier steps, will be placed on a grid with corresponding person(s) responsible. Third, we will select implementation strategies for each component of I-TRANSFER. In addition to using our teams’ wisdom, we will turn to the literature using resources such as the taxonomy of theory-based methods applicable to individual and organizational levels [70, 79, 81–83]. While the exact strategies to be employed will depend on the findings of this period of data collection, it is likely that they will consist of a multifaceted approach to implementation including staff education via webinar or in-person, changes to organizational processes and workflow, information technology, and the provision of audit and feedback to clinicians and staff taking part in the implementation study. Fourth we will produce implementation protocols, and in the fifth step we will monitor progress and fidelity during the 12-month implementation phase. While maintaining the core I-TRANSFER elements, in keeping with a pragmatic design, we will allow variation in the workflow for dyad preferences while providing monitoring and feedback of the implementation through the rollout [84]. Implementation mapping is an iterative process where steps will be revisited and modified as needed [64].

**Table 2:** Needs Assessment Objectives and Questions

Objective 1. Understand current acute care processes for identifying sepsis patients
**How are sepsis survivors identified in your hospital?**
1. Who is involved in clinical documentation and coding that the patient has/had sepsis?
o What do you do with the information? How is it shared/alerted and with whom?
2. What IT systems/tools are used?
o How are the IT systems used? (what function do they complete?)
o Criteria used to code sepsis categories?
3. How is success in identifying sepsis patients monitored?
4. What is your accuracy rate for identifying sepsis patients?
o How do you measure accuracy?
5. What are the steps in identifying sepsis patients that you think need improvement? Why?
o What is needed to achieve improvement?
6. What strategies were tried and failed?
7. What are the successful strategies?
8. What are the barriers to identification, IT documentation and communication, accuracy, monitoring accuracy?
9. To make improvements in this process, who should we work with?
10. How is sepsis defined in your hospital?
**Objective 2. Determine the workflow for referral of sepsis survivors to home health care**
What is the process for identifying sepsis survivors for referral to home health care?
1. What criteria are used to determine the need for home health referral?
2. Who is involved in making this decision?
3. When is the decision to refer the patient for home care made?
4. What IT tools are used?
5. What are the barriers to getting sepsis survivors to home health care?
6. What are some ways we could improve the process?
7. What resources are needed for improvement?
8. What are the similarities in this process across units/floors? What are the differences?
9. To make improvements in this process, who should we work with?
**Objective 3. Map the process for how and when home health is notified about the referral**
How are home health personnel notified that there is a referral to home health?
1. Who gets the notification?
2. When are they notified?
3. How are they notified?
a. Do they know the expected discharge date?
4. What IT tools are used to make the referral?
5. What are the barriers to making the referral to home health care?
6. How are the patient and family involved?
a. Who involves them?
7. We are interested in learning about areas for improvement in the process. What are some areas for improvement for the process of notifying home health of the referral?
a. What resources are needed to make improvements?
8. To make improvements in this process who should we work with?
**Objective 4. Determine what patient information is transferred during the transition to home health care**
What data elements are transferred to home health care about the referral?
1. How is the information transferred (paper, electronic, verbal)?
a. If electronic: What IT tools are used for information transfer?
b. If paper: Is this faxed or a hard copy hand off?
2. Thinking about information transfer, what challenges do you experience with transferring information from hospital to home health?
3. What makes the transfer of information easy?
4. Who sends the information and who is it intended to be received by?
5. What information is placed in the HHC record, and who uses it and for what?
6. How do nurses decide what to document as diagnoses on the OASIS?
7. Name some areas for improvement in the transfer of information and the documentation of sepsis
8. To make improvements in this process, who should we work with?
**Objective 5. Analyze the barriers and enablers of making the outpatient follow-up appointment**
How are outpatient follow-up appointments made?
1. What people are involved (hospitalist, patient, caregiver, case manager, outpatient staff, social work) in making outpatient follow up appointments?
2. How is the appointment made (what time frame, by phone, direct access)?
3. What IT tools are used to make the appointment?
4. What is the criteria for who gets an early spot on the outpatient schedule?
5. What do you do if there is no outpatient provider?
o What proportion of patients do not have an outpatient provider?
6. Would telehealth be possible if the patient does not want to make an office visit?
7. Is there a home physician/NP visiting program available?
8. How is success in making the appointment monitored?
o What proportion of the time are you successful in making the appointments?
9. What are the barriers to making the appointment within 7 days?
10. What are examples of enablers, (staff, IT, patient education, availability) to making the appointment?
11. How are patients and their caregivers involved in making follow up appointments?
12. How is the appointment communicated to the patient and/or family?
13. What are some areas for improvement in making follow up appointments?
14. What strategies were tried and failed in making follow up appointments?
15. What works?
16. Do the insurance companies play a role in facilitating early follow-up? If so, how?
17. To make improvements in this process, who should we work with?
**Objective 6. Determine the process for how home health activates timely visits**
How do you implement timely home visits (defined as: visits within 48 h of hospital discharge)?
1. What people are involved in scheduling the home health care admission visit?
2. Do the home health agency personnel know the expected hospital discharge date?
3. What conflicts arise when trying to schedule timely home health admission visits?
4. What IT tools are used?
5. What criteria is used to prioritize patient visit timing?
6. How is start of care visit timing monitored?
7. What is your current success rate for making the admission visit within 48 h of discharge?
8. What makes this difficult to do?
9. How do you think we could improve the success rate for achieving timely visits?
10. What strategies were tried and failed?
11. What works?
12. What role does geographic area play?
13. What role does the day of the week play?
14. What role does staffing play?
15. Is patient acceptance of timely visits a barrier? (just got home from hospital, don’t want you to visit)
16. To make improvements in this process, who should we work with?
**Objective 7. Determine the typical visit pattern for week one of post-acute home health care**
How are visit timing and frequency scheduled in week one for post-acute patients at home?
1. What people are involved in scheduling week one visits?
2. What IT tools are used?
3. How are the visit patterns communicated to the nurse or schedulers?
4. How is success of timing and frequency of visits during week one of care monitored?
5. What is your current visit pattern in week one for typical patients?
6. What are the barriers to achieving at least two visits in week one?
7. What are the areas for improvement?
8. What strategies were tried and failed?
9. What works?
10. To make improvements in this process, who should we work with?
**Objective 8. Explore how home health personnel can facilitate completion of the outpatient provider follow-up by one week after discharge**
Please describe the role of home health care and the processes used to encourage and help the patient keep their outpatient provider appointment?
1. Who are the people involved in encouraging the patient to attend their outpatient provider visit?
2. Who is involved in helping the patient attend the visit?
3. How is the visit attendance documented?
4. Are any IT tools used, and if yes what is used?
5. How does home health know whether or not the patient has an appointment?
6. What role does the home health provider play in obtaining an appointment for patients in the first week of home health?
7. What role does the home health provider play in facilitating attendance at outpatient appointments for patients in the first week of home health?
8. What is the current success rate for completion of timely outpatient follow-up visits?
9. How is that measured and within what time frame (7 days, 14 days)?
10. What are the barriers to the patient being seen by an outpatient provider by day 7?
11. What are the areas for improvement?
12. What strategies were tried and failed?
13. What works?
14. To make improvements in this process, who should we work with?

### Consolidated Framework for Implementation Research (CFIR) interviews

A CFIR derived interview guide that measure context, process, and implementation determinants was generated by the team using the online resources at www.CFIRguide.org. Examples include: Relative advantage: How does the intervention compare to other similar existing programs in your setting? Peer pressure: To what extent would implementing the intervention provide an advantage for your organization compared to other organizations in the area? Leadership engagement: What kind of support or actions can you expect from leaders in your organization to help make implementation successful? Patient needs and resources: How do you think individuals served by your organization will respond to the intervention? How well will the intervention meet their needs?

The team will train together in administering the CFIR interviews. CFIR interviews may be individual or in groups; implementation mapping (described above) will be done in groups as appropriate to gain consensus and promote team engagement in planning. For example, mapping the workflow for notifying the HHC intake department may be completed with the group of nurses who staff the HHC intake department. All interviews will be recorded. We will catalogue the implementation strategies and workflows suggested, adopted, modified, or discarded by the participating dyads. Data will be entered and stored in Research Electronic Data Capture (REDCap) [85, 86] or NVivo [87] as appropriate.

### Analysis

#### Aim 1, effectiveness of the I-TRANSFER intervention

A key initial task is the identification of all sepsis survivors in the national hospital dataset. Consistent with our prior work, we will use two complementary strategies to identify sepsis survivors. First, we will use the explicit International Classification of Diseases 10th Revision (ICD-10) codes (A40-41, R65.2). Second, we will employ the implicit approach used in our previous study and developed by Angus and colleagues [88] that requires a code for infection and end-organ dysfunction. Among them we will identify the sepsis survivors transitioned from participating hospitals to their partner HHC agency during the study timeframe by merging the Provider of Service File with the claim files.

Each hospital discharge of a sepsis survivor to HHC will represent a separate record on the timeline file. Some Medicare beneficiaries will have more than one index hospital discharge during the study period. Data available will give us the flexibility to define an “index” hospitalization in several ways. Our main approach will be to analyze all eligible hospitalizations followed by HHC and to adjust for the potential effect of clustering at the patient and provider level. We plan to examine the sensitivity of our results to alternative specifications of the “index” hospital stay including analyses based on a single index hospitalization per person.

Deidentified Medicare claims will be the source of information used to construct Aim 1 outcomes of rehospitalization, total hospital days if readmitted and ED utilization. The CMS claims will be stored a secure, password protected site. The CMS approach to identifying hospital readmissions was successfully used in our heart failure and sepsis studies and we will use the same approach to identify readmissions in this study [24, 50]. Observations on sepsis survivors in the baseline period will be used to check balance on covariates identified by the Andersen Behavioral Model. Any remaining imbalance will be considered by including covariates in the hierarchical regression analysis.

In addition to the survivors from the treated dyads in the baseline period, additional control survivors will be used to provide a much larger sample of control observations. These observations will be drawn from the population of Medicare sepsis survivors not being cared for by any of the participating institutions. This sample is unlikely to be balanced on covariates, a priori, with the sample of survivors from participating providers. Therefore, entropy balancing will be used to create sampling weights for these observations to produce balance on covariates. All hierarchical regression analyses will take these entropy balance weights into account. In addition to an intent-to-treat indicator, the regression will include covariates specified by the Andersen Behavioral Model. Inpatient days after the index discharge will be modeled using hierarchical Poisson regression. ED utilization will be modeled using hierarchical logistic regression.

The pragmatic aspects of the study design make it important to conduct various sensitivity and exploratory analyses. Sensitivity analyses will include checks of robustness of findings with and without sepsis survivors who are readmitted shortly after discharge (e.g., within 7 days), those with a resumption of HHC episode, discharges with very short (1 day) or very long hospital stays (30 days or more), and survivors less than 65 years old. Exploratory analyses will include estimation stratified by type of sepsis, sex (as a biological variable), and hospital ownership status.

#### Aim 2, I-TRANSFER implementation

The recorded needs assessments and CFIR interviews will be transcribed by a secure, professional service. All study materials will be stored in a secure, password protected site. The typed interview transcripts and observation notes will be uploaded into qualitative software NVivo 11.0 [50] to facilitate data organization, analysis across the research team, and documentation of steps in the workflow. The data analytic team will train together to complete the thematic analysis [89] of the transcripts and field notes. The team members will independently read through 4 randomly selected interviews taking notes on the substantive areas of content. Content identified by the research team members in this initial reading will be compared to each other and a provisional structure of the coding scheme, partially deduced from the CFIR domains, will be developed by the team [90]. The trained research assistants will complete the coding of the remaining data, meeting weekly with the investigators to review findings and confirm agreement. Ambiguities, incompleteness, lack of clarity, differences and commonalities will be discussed and resolved in weekly meetings. Alternative approaches in workflow, barriers, facilitators, and differences in acceptability observed between the study sites will be noted, compared, contrasted, and reported as study results so agencies with similar barriers can see suggested solutions to increase generalizability of the findings. NVivo 11.0 software can accommodate ongoing changes and additions to the labels describing the implementation determinants within each concept of the CFIR framework. Through constant comparative analysis, results will be refined for conceptual flow and consistency.

Strategies to increase and ensure the trustworthiness and scientific adequacy of the study include credibility (iteratively member checking the findings with the site implementation teams); and transferability (developing a thick description, notes and diagrams taken during the interviews and implementation mapping) [46]. Credibility relies heavily on input from the implementation teams and the national advisory group. Conference calls will share methods and findings with the groups to gain insights and reactions. Second, transferability or generalizability will be strengthened by collecting “thick” descriptive data that will permit comparisons with other contexts to which transfer might be contemplated. After the data collection is completed, the presentation of findings will contain a full description of contextual factors, determinants, process, and strategies so that the reader (future dyads) will be able to ascertain if they can apply the results to their situation. Third, dependability will be strengthened by conducting an audit that examines processes and strategies during implementation. Fourth, confirmability of findings will be assessed using audit and feedback and fidelity checks to determine how the workflows are implemented or modified as the dyads are onboarded [47].

An indicator on the OASIS diagnosis list documenting the identification of sepsis survivors at the beginning of HHC will be modeled using hierarchical logistic regression. We will use Medicare claims dates and site of service to identify the timing of HHC nursing visits and community provider visits. This indicator for whether the protocol for timely nursing and community provider visits was achieved will be modeled using hierarchical logistic regression. At the 12-month point from the go-live start of implementation, the study team will stop communicating and monitoring fidelity with the stakeholders. Outcomes of sepsis diagnoses and visit patterns will be compared during the “maintenance” period evaluating for stability or decline in the proportion of sepsis survivors identified and/or receiving the early visits compared to the intervention period. Hierarchical regressions will be extended through the maintenance period and separate treatment indicators for the intervention period and the maintenance period. Regressions will test equality of coefficients on the treatment indicators on the two periods. Equality of coefficients will imply maintenance of effects after the study team exits. A data monitoring committee is unnecessary as this is a low-risk, implementation science study with voluntary partners.

## Discussion

Sepsis takes more lives than opioid overdoses, breast cancer, and prostate cancer combined [4]. Prior studies by our team indicate the impact of early HHC nursing combined with early outpatient provider visits but the ability of hospitals and HHC agencies to implement these effective interventions is limited and varied. Our innovative study will be the first to transform the practice paradigm in HHC through implementation of a best practice early visit intervention for growing numbers of sepsis survivors. This study will bring process and structure to a very common transition process – currently many HHC agencies do not have the resources to study how to optimize transition of care processes, our findings will inform the industry and can apply to other disease states. The study focuses on an understudied process (transition to and care in HHC) and an understudied population (sepsis survivors) to tackle a large, costly, and common readmission challenge where little evidence exists.

Our study, however, is not without limitations. It is possible that dyads may begin to implement parts of the protocol prior to the implementation period. However, we believe the specifications of the I-TRANSFER components will be difficult to achieve in full and effectively without collaborative efforts. To be safe, additional specification checks will include treatment indicators in the retrospective control data and onboarding period to determine whether such activities were taking place. We will also ask the status and quality of such interventions prior to onboarding and would recruit a new dyad if it is an issue.

Also, the sample of sepsis survivors from non-participating dyads is a useful comparison group only if the distributions of characteristics of those survivors and their care providers overlap/match with those of survivors from the participating dyads. By using the entire Medicare population, the risk of lack of overlap is minimized. Nevertheless, should lack of overlap be an issue, we will use matching techniques to mitigate this threat. Lastly, we considered a cost analysis but due to budgetary constraints could not include a robust analysis. Instead, we will document whether there are added costs to the dyads such as adding a position, and we will have the data from the claims to roughly compare differences in service utilization such as numbers of HHC visits, outpatient care, or observation stays.

As the largest HHC study of its kind and the first to transform this type of care through implementation science, this study has the potential to produce new knowledge about the process of transition to and care in home health. If effective, the impact of this intervention during this common transition process could be widespread, improving the outcomes for a growing, vulnerable population of sepsis survivors.

## Data Availability

Per protocol, all study data will be retained by the University of Pennsylvania School of Nursing for 5 years after completion of the study. Designated research personnel, including those at Villanova University, VNS Health, and Hunter College, are granted access to the data stored in the REDCap and NVivo databases.

## Abbreviations

CCW: Chronic Conditions Warehouse
CDC: Centers for Disease Control
CFIR: Consolidated Framework for Implementation Research
CMS: Centers for Medicare and Medicaid
ED: Emergency Department
HHC: Home health care
ICD-10: International Classification of Diseases 10th Revision
I-TRANSFER: Improving Transitions and Outcomes of Sepsis Survivors
OASIS: Outcomes Assessment Information Set
ORIC: Organizational Readiness for Implementing Change
REDCap: Research Electronic Data Capture
US: United States

## Declarations

### Protocol

Issue date 2/8/2021, version 01.

Any modifications to the protocol which may impact the conduct of the study, including changes of study objectives, study design, sample sizes, study procedures, or significant administrative aspects will require a formal amendment to the protocol. Such amendment will be agreed upon by the National Institute of Nursing Research and approved by the Institutional Review Board of the University of Pennsylvania prior to implementation.

Administrative changes of the protocol are minor corrections and/or clarifications that have no effect on the way the study is to be conducted. These administrative changes will be agreed upon by the study team and will be documented in a memorandum. The Institutional Review Board of the University of Pennsylvania may be notified at the discretion of the Principal Investigator.
